# Correspondence

**Published:** 1885-04

**Authors:** J. H. Warner

**Affiliations:** Ex-Secretary and Chairman Publishing Committee, Late Ohio State Dental Society


### CORRESPONDENCE.

Columbus, O., February 4, 1885.

Editor Dental Register.—I see you devote considerable space in your January number to explaining how much money I received as Chairman of Publishing Committee, etc., during the past year. You forget to add that after paying all my bills there was a net profit of $21.00 on the transaction, which is now in the treasury of the society—the first time in the history of the State Society in which such a thing occurred and we have published them for I think nine years. Heretofore they have run the society into debt from $25.00 to $100.00 each year.

In justice to me I ask you to give this a place in your journal and oblige,

J. H. Warner,
Ex-Secretary and Chairman Publishing Committee,
Late Ohio State Dental Society.

